# Diagnosis and Management of Left Atrium Appendage Thrombosis in Atrial Fibrillation Patients Undergoing Cardioversion

**DOI:** 10.3390/medicina55090511

**Published:** 2019-08-21

**Authors:** Enrico Melillo, Giuseppe Palmiero, Adele Ferro, Paola Elvira Mocavero, Vittorio Monda, Luigi Ascione

**Affiliations:** 1 Department of Cardiology, AO dei Colli, Monaldi Hospital, 80131 Naples, Italy; 2 Institute of Biostructure and Bioimaging, National Council Research, 80131 Naples, Italy; 3 Anesthesiology and Intensive Care Unit, AO dei Colli, Monaldi Hospital, 80131 Naples, Italy

**Keywords:** atrial fibrillation, cardioversion, oral anticoagulation therapy, left atrial appendage thrombosis, transesophageal echocardiography

## Abstract

Atrial fibrillation is the most common cardiac arrhythmia and is associated with an increased risk of stroke and thromboembolic complications. A rhythm control strategy with both electrical and pharmacological cardioversion is recommended for patients with symptomatic atrial fibrillation. Anticoagulant therapy for 3–4 weeks prior to cardioversion is recommended in order to avoid thromboembolic events deriving from restoring sinus rhythm. Transesophageal echocardiography has a pivotal role in this setting, excluding the presence of left atrial appendage thrombus before cardioversion. The aim of this review is to discuss the epidemiology and risk factors for left atrial appendage thrombosis, the role of echocardiography in the decision making before cardioversion, and the efficacy of different anticoagulant regimens on the detection and treatment of left atrial appendage thrombosis.

## 1. Introduction

Atrial fibrillation (AF) is the most common sustained arrhythmia, with a prevalence of 0.4% in the general population and 9% in octogenarian patients [1]. Patients with AF have a higher risk of cardiovascular complications, including a 3–5 -old increase of stroke [2]. A 4–5 fold risk of systemic embolism (SE) [3] and a higher risk of heart failure (HF) development [4]. Without prior adequate anticoagulation, cardioversion (CV) (both electric and pharmacological) in patients with AF is associated with a non-negligible risk of thromboembolic events [5]. Consequently, for patients with AF >48 h onset, the current guidelines recommend anticoagulant therapy for at least three weeks before and four weeks after CV [6]. The transesophageal echocardiogram (TOE) is a diagnostic method that allows a detailed evaluation of the anatomy and function of the left atrial appendage (LAA), and is considered the gold standard for identifying or excluding left atrium (LA) and LAA thrombosis [6]. Vitamin K antagonists (VKAs) have traditionally been considered the gold standard for thromboembolic prophylaxis before CV; however, novel oral anticoagulants (NOACs) have also been studied, and are increasingly employed in this setting [7,8,9]. The aim of this review is to discuss the risk factors and diagnostic modalities of LAA thrombosis in AF patients undergoing CV, and to provide a summary and update of the therapeutic strategies to prevent and resolve LAA thrombosis.

## 2. Risk Factors of Left Atrial Appendage Thrombosis

LAA is the most prominent site of LA thrombus formation, with more than 90% of thrombi generating within this anatomical structure [10]. Extra-appendage thrombosis is a very rare finding in non-valvular AF and, when present, an LAA thrombus is usually concomitant [11]. The CHA_2_DS_2_-VASc score incorporates the more common stroke risk factors seen in everyday clinical practice, and is recommended to guide anticoagulant therapy in AF patients. CHA_2_DS_2_-VASc and older CHADS_2_ scoring systems are also good predictors of LAA thrombosis. CHA_2_DS_2_-VASc showed a higher sensibility and specificity for LAA thrombus detection, and severe impairment of left ventricle systolic function is a powerful predictor of LAA thrombosis [12,13,14]. Moreover, the addition of the AF type (persistent AF) and renal function to the CHA_2_DS_2_-VASc score may better stratify thromboembolic risk and could identify patients who do not need preprocedural TOE [15]. In AF patients with low thromboembolic risk, the CHA_2_DS_2_-VASc score seems more able to identify low-risk individuals with a low probability of LAA thrombus, as seen in two retrospective studies totaling 1100 AF patients where no LAA thrombi were identified in individuals with a CHA_2_DS_2_-VASc score <2 [16,17]. Conversely, a low CHADS_2_ score is less reliable in predicting the risk of LAA thrombus formation [18].

Moreover, the addition of biomarkers such as brain natriuretic peptide can improve the risk stratification of CHADS2 and CHA_2_DS_2_-VASc scores for LAA thrombus [19,20].

## 3. The Role of Echocardiography before Cardioversion

### 3.1. Transthoracic Echocardiography

Transthoracic echocardiography (TTE) should be performed for the clinical evaluation of every AF patient. TTE provides a comprehensive evaluation of cardiac anatomy and function that can help to define the type of AF and to identify patients with high risk of LAA thrombosis. Firstly, echocardiographic assessment is necessary to diagnose valvular AF, defined by the presence of moderate–severe mitral stenosis or a prosthetic valve, which is necessarily treated with VKAs anticoagulant therapy [6]. TTE provides an overall assessment of left ventricle ejection fraction (LVEF), a risk marker of both stroke and LAA thrombus presence before CV [21]. An LVEF < 40% is considered an equivalent of the congestive HF criterion in the CHA_2_DS_2_-VASc score, while a normal LVEF has been associated with a low prevalence of LAA thrombosis in AF patients undergoing TOE [22]. Evaluation of LA dimension is fundamental in assessing risk of LAA thrombosis and the probability of successful rhythm control [23]. The measurement of antero-posterior LA diameter has been traditionally considered the gold standard for LA dimension assessment. However, growing evidence suggests that the LA volume index (LAVI) is a more powerful predictor of LAA thrombus. [22]. Furthermore, a combination of LVEF-to-LAVI ratio <1.5 showed a 100% sensitivity in predicting the presence of LAA thrombus, therefore identifying a low-risk population before CV [24]. 

TTE provides markers to predict the probability of successful rhythm control before CV. LAVI, LVEF, diastolic function, E/è wave ratio, and LV hypertrophy can influence the outcome of rhythm control strategy [25,26]. The HATCH score (which stands for hypertension, age 75 years, thromboembolic event, pulmonary disease, and HF) summarizes the main markers affecting the likelihood of a successful CV [27].

The assessment of LA function with 2D speckle tracking echocardiography (STE) is a promising tool in the clinical evaluation of AF patients. Normal LA strain analysis with STE has three phases: active filling phase (reservoir), passive emptying phase (conduit), and booster pump phase (pump), which corresponds to LA contraction at the end of the ventricular diastole. In AF patients, the booster pump phase is missing, and the most reliable parameter is the peak systolic reservoir strain. STE deformation analysis is highly correlated with the amount of LA interstitial fibrosis and remodeling process occurring in AF patients [28]. A reduced peak positive strain has been associated with lower LAA emptying velocities, with an LA prothrombotic state, and with higher incidence of LAA thrombosis [29,30].

### 3.2. Transesophageal Echocardiography and Intracardiac Echocardiography

**Figure 1:**
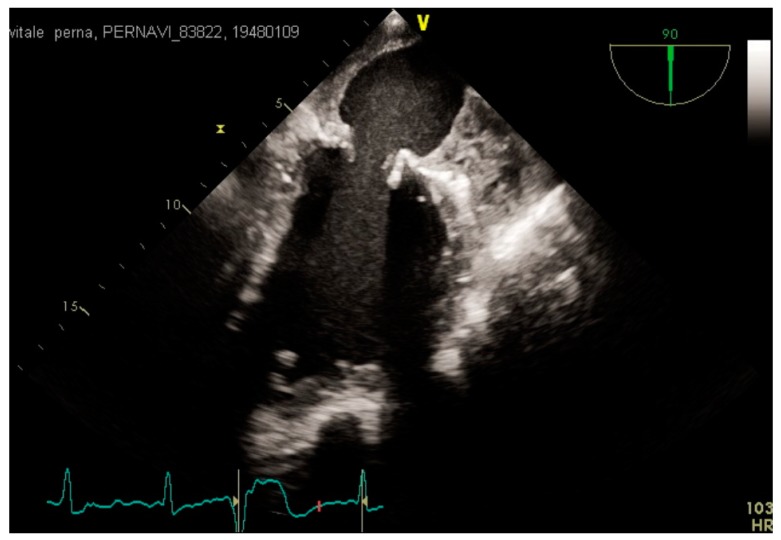
Transesophageal echocardiogram (TOE) intercommissural view showing massive left atrium (LA) and left atrial appendage (LAA) thrombosis.

**Figure 2:**
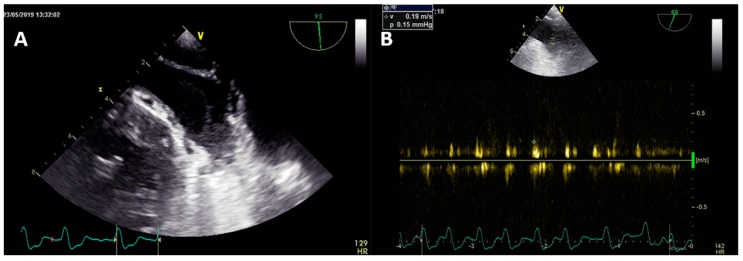
Detection of sludge in LAA with swirling effect (**A**) and associated low LAA emptying velocities (**B**).

TOE is considered the gold standard modality for diagnosis of LAA thrombi with a sensitivity and specificity of 95%–100% [31]. Current guidelines recommend TOE as an alternative to periprocedural anticoagulation in patients with >48 h AF duration or when the exact duration and onset of AF cannot be determined [6]. Moreover, in patients where thrombosis is identified, a repeat TOE after 3–4 weeks of anticoagulation should be considered before CV [6]. Modern multiplane TOE enables a visual assessment of LAA thrombi (Figure 1) or other potential intracardiac sources of embolism. TOE allows visual diagnosis of spontaneous echo contrast (SEC), also called “smoke” (Figure 2B), and “sludge” (Figure 2A), a dense and marked SEC, which is a precursor of thrombus formation and has a greater prognostic significance than smoke alone [32]. LAA mechanical function can be assessed from TOE with pulsed wave Doppler sample volume placed 1 cm below LAA ostium. LAA emptying velocities <20 cm/s are associated with a higher prevalence of SEC and LAA thrombosis [33] (Figure 2B). A combined use of color and pulsed-wave Doppler with contrast echocardiography can provide incremental information in aiding the diagnosis of LAA thrombosis in patients with doubtful diagnosis [34].

**Figure 3:**
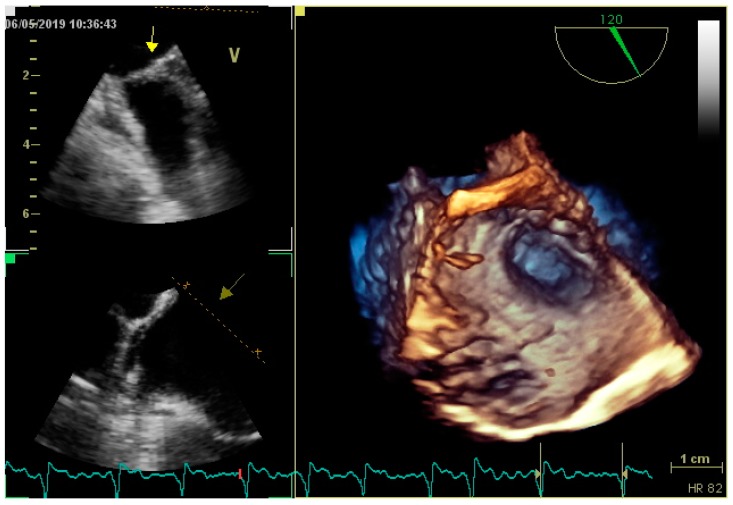
A three-dimensional TOE imaging of LAA.

However, despite the high sensitivity for thrombus detection, there are some potential limitations of the traditional bidimensional TOE. TOE may misdiagnose thrombi <2 mm, which have high embolic potential, especially in the setting of complex multilobed LAA anatomy [35]. Moreover, the LAA has a complex and highly variable three-dimensional morphology, which can render its assessment difficult using 2D TOE alone. In particular, the presence of pectinate muscles, multilobed appendage morphology, SEC, or acoustic shadowing from the Coumadin ridge might be misinterpreted as thrombi, and could prevent the accurate evaluation of the LAA with traditional 2D imaging [36]. 3D TOE improves the evaluation of LAA anatomy, overcoming some limitations associated with 2D imaging. 3D TOE enables a multiplanar reconstruction that provides a more extensive evaluation of the LAA (especially of complex multilobe morphologies), and a better depiction of the surrounding anatomical landmarks [37] (Figure 3). There is a lack of evidence regarding the sensitivity and specificity of 3D TOE for detecting LAA thrombosis. However, a recent report suggests that 3D TOE should be suggested when the diagnosis of LAA thrombosis remains equivocal after a detailed 2D analysis [36]. Moreover, with the widespread diffusion of LAA percutaneous closure techniques and increasing operator experience in 3D imaging, it is reasonable that 3D TOE will have a growing role in the diagnosis of LAA thrombosis.

Finally, intracardiac echocardiography (ICE) is an alternative imaging method of the LAA, usually indicated when TOE is contraindicated or not obtainable. ICE is performed with an 8–10 Fr catheter introduced through femoral venous access and advanced in right heart chambers under fluoroscopic guidance [38]. In the absence of interatrial transseptal crossing, the LAA can be displayed indirectly through the right ventricle outflow tract and the pulmonary artery, considering their close anatomical relationship [39]. Although ICE is less sensitive than TOE for thrombus detection [40], it can serve as a complementary method—especially when equivocal TOE findings require further evaluation. However, considering its invasive nature, in practice ICE is mainly performed in the catheterization laboratory during planned interventional cardiac procedures and when TOE is contraindicated.

## 4. Prevention and Treatment of Left Atrial Appendage Thrombosis

CV is associated with an increased risk of thromboembolic events and strokes. For patients with AF >48 h onset, current guidelines recommend anticoagulant therapy for at least three weeks before and four weeks after CV [6]. The reason for three weeks of anticoagulation before electrical CV derives from a study suggesting that at least 14 days are needed for fibroblastic infiltration and organization of an LAA thrombus [41]. The four weeks of anticoagulation after CV are due to postprocedural LA stunning, with marked impairment of Doppler-derived indexes of LA contraction that contribute to a higher risk of thromboembolic stroke [5,42]. 

VKAs are considered the gold standard for thromboembolic prophylaxis before CV, and are the only recommended treatment in patients with valvular AF [6]. Previous studies suggest that the incidence of LA/LAA thrombosis under treatment with VKAs ranges between 0.6% and 7%, depending on population study and sample size [43,44,45]. The narrow therapeutic interval and difficulties in keeping a target time in therapeutic range are considered the main limitations of VKA therapy. 

NOACs are safe and effective alternatives to VKA therapy [46,47,48,49], and are currently considered as first-line therapy for long-term stroke prevention in patients with nonvalvular AF [6]. The overall safety and efficacy profile of NOACs have been confirmed in different clinical scenarios [50,51,52,53,54,55,56,57,58]. The clinical performance of NOACs in the setting of acute and elective CV has been addressed by three prospective randomized trials. In the X-VeRT trial, 1504 patients with AF of either >48 h or unknown duration undergoing CV were randomized to once-daily rivaroxaban or VKA [7]. The composite endpoint of stroke or systemic embolism after CV occurred in 1.02% of patients on VKAs and in 0.51% of patients on rivaroxaban. More recently, the ENSURE-AF trial randomized 2199 patients with AF >48 h and <1 year duration undergoing electrical cardioversion to 60 mg edoxaban once daily or warfarin with enoxaparin bridging [8]. The primary efficacy composite endpoint of stroke, systemic embolic event, myocardial infarction, and cardiovascular mortality at 28 days post CV occurred in 1.0% of patients of both arms. The EMANATE trial randomized 1500 patients with AF of ≤48 h onset to apixaban or heparin/VKA [9]. The trial showed a significant reduction with apixaban in strokes (0% vs. 0.8%) and in major bleeding (0.4% vs. 0.8%) at 30 days compared to warfarin. The favorable clinical profile of NOACs in patients undergoing CV has also been confirmed in a recent metanalysis [59,60] and in real-world studies [61,62,63,64,65,66].

Probably as a result of this evidence, a recent multicentric European registry addressing NOAC strategies before CV showed a trend towards an increasing use of NOACs and a significant decrease of VKA use [67]. However, in this registry 68.5% of patients received VKA anticoagulant therapy before CV and clinicians appeared to hesitate to embrace NOAC usage before CV [67].

In a real-world clinical scenario, the average time to CV was shorter with NOACs compared to warfarin [62]. Moreover, a budget impact analysis showed that the potential use of an early rivaroxaban strategy before direct-current CV could lead to a significant saving of costs related to procedure [68]. Therefore, considering these positive points and the effective clinical profile versus warfarin, it is reasonable that the use of the NOAC anticoagulation strategy before CV could increase in coming years.

Although randomized trials have addressed the efficacy and clinical profile of NOACs versus warfarin before CV, the prevalence of LA/LAA thrombus after adequate anticoagulation with NOACs remains a relatively unaddressed issue. In the X-VeRT study, a relatively high occurrence of LAA thrombi (18.2%) was observed in the 33 patients enrolled in the trial who underwent TOE before elective CV [7]. However, only about 10% of patients scheduled for elective CV in the X-VeRT trial underwent TOE, and these data could explain the high percentage of LAA thrombosis. In the ENSURE-AF trial, 47 patients (8%) in the edoxaban arm and 42 patients (7.1%) in the enoxaparin-warfarin arm had LAA thrombosis on pre-CV TOE [8]. In the EMANATE trial, LA/LAA thrombus was detected in 61 patients (4%), 30 in the apixaban group, and 31 in the heparin/VKA group, in over 829 patients who underwent TOE before CV [9]. 

There is a paucity of data on the prevalence of LAA thrombosis on NOAC therapy before CV in a real-life setting. A multicenter real-world study performed in AF patients undergoing TOE 12 h before CV or catheter ablation reported LAA thrombus in 3.6% of patients (15/414) with no significant difference between dabigatran, rivaroxaban, and apixaban [69]. Another recent real-world study showed LAA thrombosis in 7/127 patients (5.5%) and SEC in 24/127 (18.9%) [70].

Therefore, these data firstly suggest that LAA thrombosis is not a negligible event despite adequate anticoagulation, and that—although not universally accepted—a TOE-guided CV strategy is mandatory in order to reduce the burden of thromboembolic periprocedural events and to reduce bleeding events [71].

Once an LAA thrombus is identified, effective anticoagulation is recommended for at least three weeks or until LAA thrombus resolution is detected on follow-up TOE [6]. In this setting, VKA therapy for four weeks with repeat TOE showed resolution of thrombi in 80%–89% patients who were not previously anticoagulated [72,73]. If LAA thrombus occurs despite therapeutic anticoagulation, different strategies can be pursued, including switching to NOACs. Data are lacking on LAA resolution after NOACs therapy, and principally comprise case reports, where NOACs were initiated after failure of VKAs therapy or low time in therapeutic range [74,75,76]. The X-TRA multicenter prospective trial explored the use of rivaroxaban for the treatment of firstly-diagnosed LAA thrombus in 53 patients with available baseline and follow-up TOE [77]. About 75% of the patients had no prior anticoagulant therapy, while approximately one-quarter were treated with subtherapeutic VKA therapy. After six weeks of treatment, repeat TOE found a resolution of LAA thrombi in 41.5% of patients, 19% had LAA thrombi reduced in size, while 17% were unchanged and 22.5% had an increase in thrombi size [77]. 

In a recent retrospective study, among the 1485 patients with AF undergoing TOE, LAA thrombus or sludge was detected in 117 patients (7.8%) [78]. Of these, 39 (33%) were prescribed an NOAC, with rivaroxaban being the most frequently prescribed (54%). On repeat TOE, LAA thrombus resolution was seen in 37 patients (58.7%) with higher resolution rates, although not statistically significant, with NOAC therapy. A recent report on TOE showed 4.7% of LA/LAA thrombosis in 864 AF patients [79]. Follow-up TOE was performed in 22 patients, and 19 of them had LA/LAA thrombus resolution. In this real-world study, the preferred anticoagulant strategy was an uptitration of NOAC dosages and keeping higher INR values for warfarin therapy. However, a reduced NOAC dosage was most frequently associated with LA/LAA thrombi detection [79].

In conclusion, there is still a lack of solid evidence on the efficacy and safety of NOACs in LAA thrombus resolution. Current ongoing randomized trials comparing NOACs with VKAs in LA/LAA thrombosis resolution (rivaroxaban NCT03792152; dabigatran NCT02256683) will provide further evidence in this clinical scenario.

## 5. Conclusions

- The incidence of LAA thrombosis in AF patients undergoing CV is not a negligible event despite adequate anticoagulant therapy.
- A TOE-guided CV is a mandatory strategy in order to reduce the burden of periprocedural thromboembolic events.
- Although VKAs have been historically considered the cornerstone anticoagulant therapy before CV, growing evidence show that NOACs are safe and effective alternatives in this setting.
- Further and extended data are needed to assess the efficacy and safety profile of NOACs for the treatment of LAA thrombosis.
